# The Tomato lncRNA47258-miR319b-TCP Module in Biocontrol Bacteria Sneb821 Induced Plants Resistance to *Meloidogyne incognita*

**DOI:** 10.3390/pathogens14030256

**Published:** 2025-03-05

**Authors:** Fan Yang, Xiaoxiao Wu, Lijie Chen, Mingfang Qi

**Affiliations:** 1 College of Horticulture, Shenyang Agricultural University, Shenyang 110866, China; yangfan20241107@163.com; 2 College of Plant Protection, Shenyang Agricultural University, Shenyang 110866, China; wuxiaoxiao1233@163.com

**Keywords:** lncRNA, nematode, tomato, biocontrol bacteria

## Abstract

Long non-coding RNAs (lncRNAs) represent a class of non-coding RNAs. In the study of *Pseudomonas putida* Sneb821-induced tomato resistance to *Meloidogyne incognita*, reverse transcription polymerase chain reaction (RT-PCR) was employed to validate 12 lncRNAs in tomato. Among them, the lncRNA47258/miR319b/TCP molecular regulatory module was likely implicated in the process of Sneb821-induced tomato resistance against *M. incognita*. Through the application of tomato hairy root and virus-induced gene silencing (VIGS) technologies for the investigation of lncRNA47258, it was determined that lncRNA47258 could target the *TCP* (*Solyc07g062681.1*) gene and modulate the metabolic pathway of tomato jasmonic acid-related indices, thereby impeding the infection of *M. incognita*. Moreover, the overexpression of the target gene *TCP* (*Solyc07g062681.1*) using tomato hairy root technology demonstrated that it could regulate the jasmonic acid synthesis pathway in tomato, consequently obstructing the infection and suppressing the development of *M. incognita*. Collectively, lncRNA47258/miR319b/*TCP* (*Solyc07g062681.1*) was preliminarily verified to be involved in the Sneb821-induced resistance process against *M. incognita* in tomato.

## 1. Introduction

In agriculture, root-knot nematodes (RKN) are the most widespread and economically damaging pest. These obligate plant-parasitic nematodes, commonly known as RKN, reproduce and feed within plant roots, causing galls or root knots. With a global distribution, RKN infects about 2000 plant species across diverse habitats, contributing to around 5% of global crop losses. The genus RKN includes over ninety species, but *M. incognita* is considered one of the most significant worldwide, and one of the key pests of tomato [1]. The plant hormone jasmonic acid (JA) and its derivatives, like methyl jasmonate (MeJA), play a key role across the plant kingdom. In tomatoes, JA or its derivatives serve as long-distance wound signals. Systemin, a specific inducer of protein inhibitors (PIs), is essential for JA synthesis during insect and nematode attacks and wounding. JA was also found to enhance tomato resistance against root-knot nematodes [2].

Long non-coding RNAs (lncRNAs) represent a class of non-coding RNAs with a length exceeding 200 nucleotides. They can interact with miRNAs and thereby influence gene expression [3]. LncRNAs have been demonstrated to play essential roles in numerous plant biological processes, such as plant pathogen resistance [4]. Functional assays revealed that seedlings with silenced GhlncNAT-ANX2 and GhlncNAT-RLP7 exhibited enhanced resistance to pathogenic fungi, potentially due to the elevated expression of LOX1 and LOX2 [5]. Previous investigations have indicated that lncRNA (DRIR) exerts a positive regulatory effect on drought and salt stress, and Arabidopsis mutants display robust stress tolerance to drought and salt [6]. The overexpression of lncRNA-At5NC056820 in Arabidopsis can enhance the drought tolerance of Arabidopsis to a certain degree [7]. Through lncRNA differential expression and statistical analysis, Li identified four gibberellin-responsive lncRNAs (GARR1, 2, 3, and 4) in wild-type and GA-sensitive dwarf maize, and determined that GARR2 originated from a Gypsy transposon [8]. LncRNA15492 was found to be involved in the interaction between tomato and bacteria [9]. The lncRNA23468/miR482b/NBS-LRR and WRKY1/lncRNA33732/RBOH models could modulate the accumulation of H2O2 in tomato to enhance its disease resistance [10]. Previous studies have verified that the CYP380C6, CYP4CJ1, CYP6DA2, CYP6CY7, and CYP6CY21 genes might participate in the formation of resistance to helix tetramer in cotton aphid. 366492/5, and MSTRG.71880.1 may regulate the expression of CYP6CY21 and CYP380C6 via the “sponge effect” by binding to miRNA [11]. LNC610 is implicated in the regulation of high light-induced anthocyanin production and functions as a positive regulator to promote MdACO1 gene expression and ethylene biosynthesis [12]. LncRNA7, lncRNA2, and their regulatory genes regulate the cotton cell wall defense against Verticillium wilt through auxin-mediated signal transduction, presenting a novel strategic foundation for cotton breeding [13]. The effect of lncRNA354-miR160b on the expression of GhARF17/18 may regulate auxin signaling, consequently affecting plant growth. This study uncovers a lncRNA-related salt stress response mechanism and also reveals that the lncRNA-miRNA-mRNA regulatory module can effectively respond to plant stress resistance [14]. In tomato, a large number of lncRNAs are engaged in the host defense response against potato spindle tuber viroids [15]. Another gene, lncRNA0957, was induced by virus in susceptible tomato plants to augment tomato resistance to etiolated leaf warp virus [16]. Additionally, siRNAs between tomato etiolated leaf virus genes were transferred to tomato plants to target host lncRNAs, thereby modulating disease symptoms [17]. Our previous studies discovered that some miRNAs and lncRNAs might stimulate tomato immunity in response to *P. putida* to resist *M. incognita* infection, and lncRNA47258 may be a key factor in the process of biocontrol bacteria-induced tomato resistance to RKN [18]. These findings offer a novel molecular regulatory model for the mechanism of bacteria-induced plant resistance to *M. incognita* infection in tomato.

## 2. Materials and Methods

### 2.1. Species, Plants, and Nematodes Tested

Strain Sneb821 of *P. putida* and the Moneymaker of tomato varieties were deposited by the Northern Institute of Nematodes, Shenyang Agricultural University. *M. incognita* was bred in a greenhouse at the Northern Nematode Institute, Shenyang Agricultural University.

### 2.2. Materials for Test

pCAMBIA1302, purchased from Beijing Zhuang Meng International Biological Gene Technology Co., LTD.; TRV1 and TRV2 were presented by Shenyang Agricultural University. QuickCut™ Xho I restriction enzyme, purchased from TaKaRa (Dalian, China); A. tumefaciens GV3101, purchased from Shanghai Wedi Biotechnology.

### 2.3. Total RNA Extraction, Reverse Transcription cDNA, and qPCR Analysis of Tomato

The specific method was referred to in our previous study [18,19].

### 2.4. Amplification of Target Fragments

The instructions for TaKaRa PrimeSTAR Max DNA Polymerase were followed.

Refer to our previous study [18]. The TRV2 vector was digested with Xho I and BamH I, and the target gene fragment was reversed into the TRV2 vector. pCAMBIA1302 was digested using Xho I.

### 2.5. E. coli Transformation and Culture of Tomato Hairy Roots

The specific method was referred to in our previous study [18,19].

### 2.6. Determination of Jasmonic Acid Content

After precooling at −80 °C in the mortar used, the test material stored in an ultra-low temperature refrigerator was removed. Samples were preheated with a small amount of liquid nitrogen, then a small amount of liquid nitrogen was added and ground in an ice bath. Add 4 mL of 80% methanol as the extract solution (the extract solution needs to be precooled at −20 °C), ground into a uniform centrifuge tube in an ice bath, and then transfer 10 mL of the sample to the refrigerator for 4 min. The centrifuge tube adds 4 mL of the extract solution to the remaining precipitate, then stir well, leach at 4 °C for 1h, and centrifuge as above. The supernatants were pooled and the residues discarded. The collected supernatant was tested through a C-18 solid-phase extraction column.

### 2.7. Nematode Infection and Enzyme Activity Detection

The specific method was referred to in our previous study [18].

### 2.8. Histochemical Staining and Tomato GUS Staining Were Performed

The specific method was referred to in our previous study [19].

### 2.9. Statistical Analysis of Data

In this study, SPSS19.0 software was used for data analysis and processing, and the Duncan method was used to analyze the significance of the data in this study, and the data results were expressed as the mean ± standard deviation. Mapping was performed using prism 8.

## 3. Results

### 3.1. RT-PCR Validation of Differential lncRNA in Tomato

Specific primers were meticulously designed, and RT-PCR was employed to authenticate the differential lncRNA within the transcriptome (Supplementary Figure S1). Lanes 1–12 corresponded to lncRNA18894, lncRNA21563, lncRNA24059, lncRNA25797, lncRNA35115, lncRNA39939, lncRNA47258, lncRNA44664, lncRNA45969, lncRNA48734, lncRNA51612, and lncRNA7183, all of which were successfully amplified in the transcriptome.

### 3.2. Expression Analysis of lncRNA47258 in Tomato

In accordance with the ceRNA (competing endogenous RNA) outcomes predicted by the preceding analysis [18], lncRNA47258 is capable of functioning as an endogenous target mimic of miR319b, thereby influencing the expression level of miR319b. The miR319/TCP4 molecular regulatory module is involved in the modulation of the jasmonate signaling pathway within tomato plants. Subsequently, it contributes to the plant’s resistance against *M. incognita* [20].

**Figure 1:**
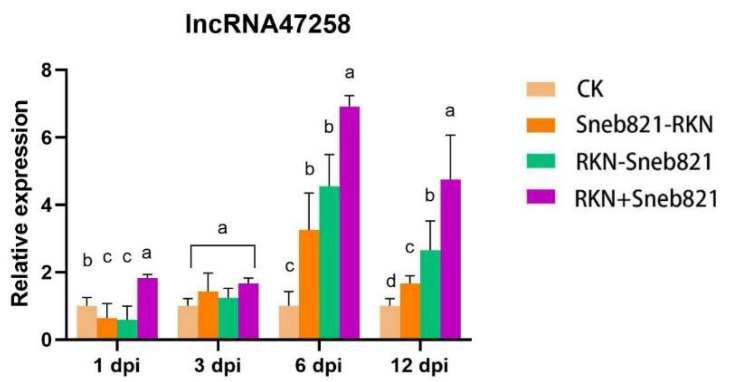
Expression levels of lncRNA47258 in each treatment at each time point. Note: CK (Control Check) represents no treatment, Sneb821-RKN represents inoculation with biocontrol bacteria Sneb821 only, RKN-Sneb821 represents inoculation with *M. incognita* only, and RKN + Sneb821 represents double treatment. The letters above the bars indicate significant difference at a value of *p* < 0.05.

In the present study, the quantitative analysis of lncRNA47258 was conducted. The results demonstrated that the expression of lncRNA47258 was remarkably elevated in tomato at the late stage (6 days post-inoculation and 12 days post-inoculation) following inoculation with nematodes and biocontrol bacteria (RKN + Sneb821). Moreover, the expression of lncRNA47258 was significantly higher in the combined treatment of biocontrol bacteria and nematodes compared to the single treatment (Sneb821-RKN or RKN-Sneb821) (Figure 1). These findings suggested that lncRNA47258 played a crucial role in the resistance of tomato to *M. incognita* infection induced by Sneb821.

### 3.3. Construction of lncRNA47258 Overexpressed Tomato Plants

lncRNA47258 was found to be overexpressed in the roots of tomato plants. Subsequently, its overexpression vector, pCAMBIA1302-lncRNA47258, was constructed. Based on the fragment sequence of lncRNA47258 within the tomato transcriptome, the genomic location of lncRNA47258 on the tomato genome is presented in Supplementary Figure S2A. Specific primers were designed to amplify the full length of lncRNA. After the PCR reaction, a band approximately 900 bp in size was obtained, which was in accordance with the length of lncRNA47258. Following purification, the amplified products were ligated with the cloning vector pMD20-T and then transformed into Escherichia coli. Positive monoclonal clones were selected for plasmid extraction. The plasmids were identified through enzyme digestion and PCR detection, and bands corresponding to the size of the target gene were obtained (Supplementary Figure S2B). The constructed plasmid pCAMBIA1302-lncRNA47258 was introduced into Agrobacterium rhizogenes MSU440 via the freeze–thaw method and subsequently transformed into tomato plants. GFP fluorescence labeling was employed to verify the transformation of positive tomato plants (Supplementary Figure S2C). Collectively, these results demonstrated that lncRNA47258 was successfully overexpressed in pCAMBIA1302-lncRNA47258 transgenic tomato plants.

### 3.4. Overexpression of Tomato lncRNA47258 Inhibited the Infection and Development of M. incognita

**Figure 2:**
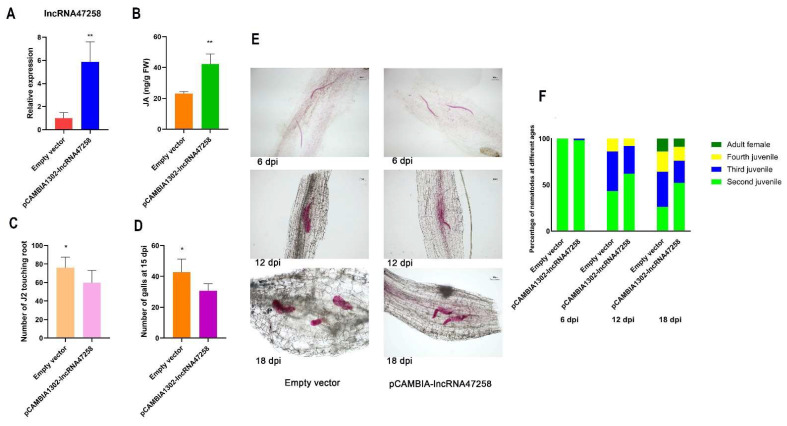
Response of lncRNA47258 to *M. incognita* infection and development. Note: (**A**) Relative expression level of lncRNA47258 in pCAMBIA1302-lncRNA47258 and control group, (**B**) JA content in pCAMBIA1302-lncRNA47258 and control group, (**C**) statistics of J2 in pCAMBIA1302-lncRNA47258 and control group, (**D**) statistics of galls number in pCAMBIA1302-lncRNA47258 and control group, (**E**) development of *M. incognita* in pCAMBIA1302-lncRNA47258 and control group, and (**F**) percentage of different ages of *M. incognita* in pCAMBIA1302-lncRNA47258 and control group. * indicate a significant difference at the *p* = 0.05 level, ** indicate a significant difference at the *p* = 0.01 level.

To investigate the potential role of lncRNA47258 in the induction and resistance of Sneb821, the expression of lncRNA was examined in tomato plants overexpressing pCAMBIA1302-lncRNA47258. Notably, lncRNA47258 expression was substantially up-regulated in these overexpressed plants (Figure 2A). Concurrently, the content of jasmonic acid exhibited a significant increase (Figure 2B), suggesting a positive correlation between lncRNA47258 and jasmonic acid synthesis in tomatoes.

The constructed pCAMBIA1302-lncRNA47258 overexpression tomato plants were inoculated with second-stage juveniles (J2) of *M. incognita*. Remarkably, the J2 infection amount in the tomato plants was considerably reduced, with approximately 15 fewer nematodes per plant (Figure 2C). At 15 days post-inoculation (dpi), the number of root knots in pCAMBIA1302-lncRNA47258 roots was significantly diminished (Figure 2D).

The development of *M. incognita* in lncRNA47258 overexpression plants and control plants was evaluated at 6 dpi, 12 dpi, and 18 dpi (Figure 2E). By calculating the percentage of nematodes at each instar at each inoculation time point, it was determined that the development of *M. incognita* in tomato plants overexpressing lncRNA47258 was delayed (Figure 2F).

Collectively, these results demonstrate that the overexpression of lncRNA47258 in tomato plants can enhance the jasmonate content in tomato roots, significantly decrease J2 infection in tomato roots, delay nematode development, and inhibit root-knot formation. These findings imply that Sneb821 can augment the jasmonate content in tomato roots by activating the expression of lncRNA47258, thereby conferring resistance against *M. incognita* infection and impeding the development of *M. incognita*.

### 3.5. Construction of lncRNA47258 VIGS Vector in Tomato

In accordance with the established sequence, the transcriptome lncRNA47258 was screened using the SGN VIGS Tool (https://vigs.solgenomics.net/, accessed on 1 May 2022) to identify the lncRNA47258 silence target fragment. Specific primers were then designed, and following PCR amplification, a band approximately 300 bp in size was obtained from the tomato variety MoneyMaker, which corresponded to the fragment length within the target gene lncRNA47258. After purification, the PCR amplification product was ligated into the cloning vector pMD20-T, yielding the product pMD20-T-lncRNA47258. This was subsequently transformed into Escherichia coli. A positive monoclonal clone was selected, and the plasmid was extracted. The plasmid was then verified through enzyme digestion and PCR analysis, with a band corresponding to the target fragment being observed. Subsequently, the lncRNA47258 fragment was ligated into the expression vector of TRV2 via homologous recombination. The results of the double enzyme digestion are presented in Supplementary Figure S3.

### 3.6. Silencing lncRNA47258 Reduces the Resistance of Tomato to M. incognita

**Figure 3:**
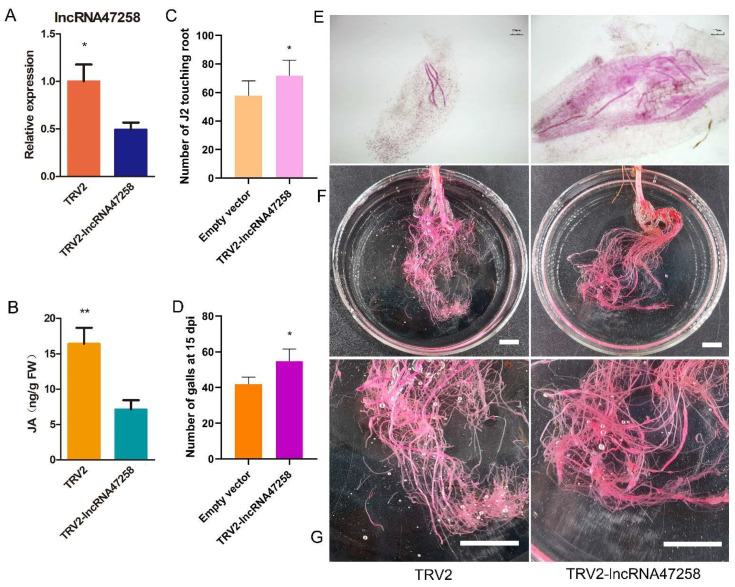
Response of VIGS-lncRNA47258 to *M. incognita* infection. Note: (**A**) Relative expression level of lncRNA47258 in TRV2-lncRNA47258 and control group, (**B**) JA content in TRV2-lncRNA47258 and control group, (**C**) J2 statistics in TRV2-lncRNA47258 and control group, (**D**) statistics of galls number in TRV2-lncRNA47258 and control group, (**E**) infection of J2 in TRV2-lncRNA47258 and the control group, and (**F**,**G**) galls situation in TRV2-lncRNA47258 and control group (E scale bars = 100 μm, F and G scale bars = 1 cm). * indicate a significant difference at the *p* = 0.05 level, ** indicate a significant difference at the *p* = 0.01 level.

To investigate the function of lncRNA47258 in tomato plants, a virus-induced gene silencing (VIGS) system was established to suppress its expression. After 21 days of VIGS treatment, quantitative real-time polymerase chain reaction (qRT-PCR) analysis revealed a significant reduction in lncRNA47258 expression in the silenced tomato plants. The expression level of lncRNA47258 was approximately 50% lower compared to that in TRV2 control plants (Figure 3A). The TRV2-lncRNA47258 silenced tomato plants were then utilized to determine the jasmonic acid content. Notably, the jasmonic acid content in the roots of TRV2-lncRNA47258 plants was substantially decreased compared to TRV2 plants (Figure 3B). Six days post-inoculation with *M. incognita*, the number of J2 nematodes infecting the roots was quantified. The results demonstrated a significant increase in J2 infection in TRV2-lncRNA47258 tomato plants, with approximately 10 more J2 nematodes per plant root (Figure 3C,E). Additionally, at 15 days post-inoculation (dpi), the number of root knots in pBI121-lncRNA48734 roots was remarkably elevated (Figure 3D,F,G). These findings imply that the silencing of lncRNA47258 in tomato plants leads to an enhanced J2 infection in the roots. In conjunction with the expression analysis results of lncRNA48734, it is hypothesized that lncRNA48734 exerts a negative regulatory role during *M. incognita* infection. The silencing of lncRNA47258 might inhibit jasmonic acid expression, consequently influencing the resistance of tomato plants against *M. incognita*.

### 3.7. Verification of Target Sites of Tomato lncRNA47258 and Target Gene TCP (Solyc07g062681.1)

**Figure 4:**
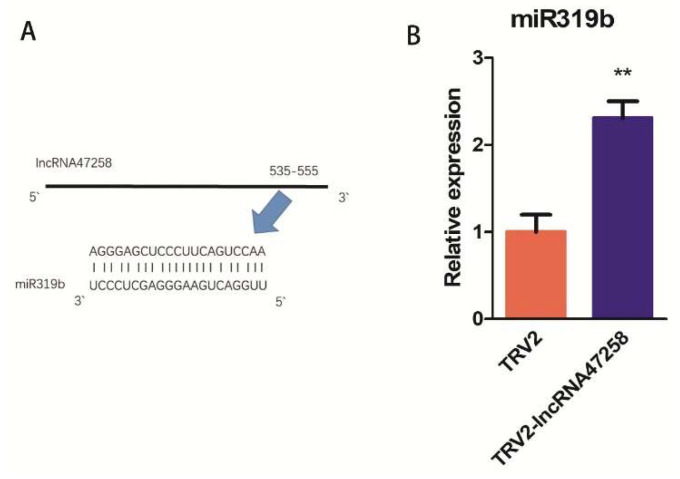
lncRNA47258 contained eTM of miR319b. Note: (**A**) The eTM binding site of lncRNA47258 to miR319b, and (**B**) relative expression level of miR319b in TRV2-lncRNA47258 and control group. ** indicate a significant difference at the *p* = 0.01 level.

Endogenous target mimics (eTMs) of miRNA on lncRNA enable lncRNA to impede the adsorption of miRNA, thereby inhibiting miRNA expression and subsequently influencing the expression of its target genes. The eTM binding site of miR319b was previously predicted within lncRNA47258, and the specific site position was determined to be from 535 nt to 555 nt on lncRNA (Figure 4A). Moreover, compared with TRV2 tomato plants, the expression of miR319b was remarkably up-regulated in TRV2-lncRNA47258 tomato plants (Figure 4B), suggesting that lncRNA47258 functions as an endogenous mimic target of miR319b.

**Figure 5:**
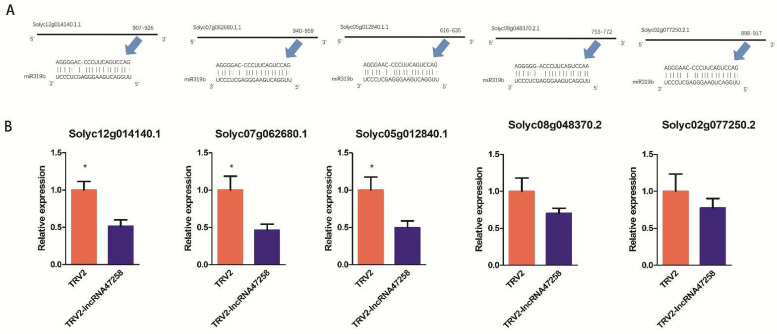
Expression level of TCP gene in tomato plants after silencing the lncRNA47258 gene. Note: (**A**) The eTM binding site of TCP genes to miR319b, and (**B**) relative expression level of TCP genes in TRV2-lncRNA47258 and control group. * indicate a significant difference at the *p* = 0.05 level.

The TCP gene serves as a promoting factor for cell proliferation in plants. In rice, the overexpression of the TCP19 gene associated with miR319 can result in the high expression of genes related to multiple signaling pathways, such as the jasmonic acid and auxin pathways [21]. The genes Solyc12g014141.1, Solyc07g062681.1, and Solyc05g012840.1 were investigated. The binding sites of miR319b to these three TCP genes were predicted and subsequently confirmed (Figure 5A). Additionally, the expression of these three TCP genes in TRV2-lncRNA47258 tomato plants was examined, and it was found that the expression of these three genes was significantly down-regulated in comparison with that in TRV2 plants (Figure 5B). These findings suggest that the expression of the tomato TCP gene is regulated by lncRNA47258.

### 3.8. Construction of Tomato Plants Overexpressing TCP (Solyc07g062681.1), the Target Gene of lncRNA47258

The target gene of lncRNA47258 was identified as TCP (Solyc07g062681.1). Notably, the expression level of TCP was significantly up-regulated in the biocontrol treatment (Supplementary Figure S4A). Subsequently, to further investigate the function of TCP, it was overexpressed in tomato roots. The overexpression vector pCAMBIA1302-Solyc07g062680.1 was constructed. Specific primers were meticulously designed to amplify the full length of the TCP gene. After the PCR reaction, a distinct 1200 bp band was obtained, which precisely corresponded to the expected length of the target gene. Following purification, the amplified products were ligated with the cloning vector pMD20-T and then transformed into Escherichia coli. Positive monoclonal clones were selected for plasmid extraction. The plasmids were verified through enzyme digestion and PCR detection, and bands consistent with the size of the target gene were successfully obtained (Supplementary Figure S4B). The constructed plasmid pCAMBIA1302-Solyc07g062680.1 was introduced into Agrobacterium rhizogenes MSU440 via the freeze–thaw method and subsequently transformed into tomato plants. The transformed tomato plants were validated by GFP fluorescent labeling (Supplementary Figure S4C), thereby confirming the successful overexpression of TCP in tomato plants.

### 3.9. Overexpression of Tomato TCP (Solyc07g062680.1) Inhibited the Infection and Development of M. incognita

**Figure 6:**
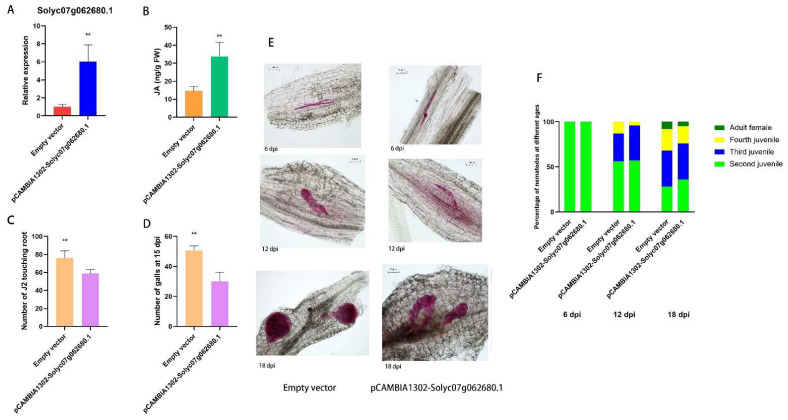
Response of TCP (Solyc07g062680.1) to *M. incognita* infection and development. Note: (**A**) Relative expression level of Solyc07g062680.1 in pCAMBIA1302-Solyc07g062680.1 and control group, (**B**) JA content in pCAMBIA1302-Solyc07g062680.1 and control group, (**C**) statistics of J2 in pCAMBIA1302-Solyc07g062680.1 and control group, (**D**) statistics of galls number in pCAMBIA1302-Solyc07g062680.1 and control group, (**E**) development of *M. incognita* in pCAMBIA1302-Solyc07g062680.1 and control group, and (**F**) percentage of different ages of *M. incognita* in pCAMBIA1302-Solyc07g062680.1 and control group. ** indicate a significant difference at the *p* = 0.01 level.

The expression of pCAMBIA1302-Solyc07g062680.1 was found to be remarkably up-regulated in tomato plants with pCAMBIA1302-Solyc07g062680.1 overexpression (Figure 6A). Concomitantly, the content of jasmonic acid was also significantly elevated (Figure 6B), suggesting a positive correlation between TCP and jasmonic acid synthesis in tomato. The constructed pCAMBIA1302-Solyc07g062680.1 overexpressing tomato plants were inoculated with *M. incognita* J2. At 6 days post-inoculation (dpi), the J2 infection in tomato plants was substantially reduced, with approximately 15 fewer J2 per tomato plant (Figure 6C). At 15 dpi, the number of root knots in pCAMBIA1302-Solyc07g062680.1 roots was significantly diminished (Figure 6D). The development of TCP overexpression plants and control *M. incognita* was quantified at 6 dpi, 12 dpi, and 18 dpi (Figure 6E), and the percentage of nematodes at each instar at each time point was calculated (Figure 6F). It was observed that the overexpression of TCP led to a delay in the development of *M. incognita* in tomato roots. Collectively, these results demonstrated that the overexpression of TCP in tomato plants could enhance the jasmonic acid content in tomato roots and significantly mitigate the J2 infection in tomato roots. This indicates that Sneb821 modulates the target gene TCP to augment the jasmonic acid content in tomato roots by activating the expression of lncRNA47258, thereby conferring resistance against infection and inhibiting the development of *M. incognita*.

## 4. Discussion

Root-knot nematode disease ranks among the most severe soil-borne diseases of crops globally, leading to substantial economic losses in tomato production. Biological control, as an effective control strategy, has garnered increasing attention. Non-coding RNA, which comprises small RNA molecules not encoding proteins, has emerged as a novel research focus in molecular biology. The mechanism underlying ceRNA (endogenous competing RNA) in plant resistance to diseases induced by bio-control bacteria represents an urgent research gap. Previous investigations have demonstrated that Slylnc0195, acting as a “sponge” for miR166, can disrupt the expression of the HD-Zip TF gene in tomato etiolated leaf warp virus disease. The ceRNA network of the Sly-lnc0195-miR166-HD-Zip TF gene can modulate the expression of its target protein HD-Zip TF, thereby influencing tomato’s resistance to external diseases [22]. In the study of tomato late blight, a set of ceRNAs, lncRNA23468-miR482b-NBSLRR, has been identified. When lncRNA23468 is overexpressed in tomato, the expression of miR482b is markedly reduced, and the expression of its target gene NBS-LRR is enhanced. These outcomes enhance the resistance of tomato plants to Phytophthora infestans. Conversely, silencing lncRNA23468 results in an elevation in the expression of miR482b and a decline in the expression of NBS-LRR, leading to a reduction in the resistance of tomato plants to the pathogenic phytophthora [23].

In the present study, tomato plants were treated with *P. putida* Sneb821 and inoculated with *M. incognita*. Whole-transcriptome sequencing analysis was conducted, and differentially expressed non-coding RNAs were analyzed and screened. Subsequently, the ceRNA regulatory network module among lncRNA-miRNA-mRNA was constructed. Moreover, gene overexpression and VIGS were employed to validate the mechanism of non-coding RNA and its target genes during tomato resistance to *M. incognita*, aiming to furnish a theoretical foundation for the biological control of root-knot nematodes. The key findings are summarized as follows.

In recent years, it has been revealed that lncRNA, as a novel regulator, can influence miRNA expression via endogenous mimetic target eTM sites within gene sequences [17]. The eTMs containing miR166 and miR399 in tomato slylnc0195 and slylnc1077 affect tomato resistance to etiolated leaf virus by suppressing the expression of miRNA [21]. lncRNA23468, lncRNA13262, and lncRNA01308 possess miR482b eTM. The overexpression of lncRNA23468 in tomato diminishes the expression level of miR482b and augments resistance to late blight. Silencing lncRNA23468 elevates the expression level of miR482b and plant susceptibility to late blight [24].

In this study, lncRNA47258 was predicted to be an eTM for miR319b to adsorb miRNA. The silenced tomato plants exhibited the up-regulated expression of miR319b, a decreased jasmonic acid level, an inhibited expression of the corresponding three TCP target genes, and reduced resistance to *M. incognita*. In contrast, the overexpression of lncRNA47258 increased the jasmonic acid level, up-regulated TCP target genes, and decreased nematode infection and root-knot number. Our results suggest that Sneb821 regulates tomato jasmonic acid synthesis through the lncRNA47258/miR319b/TCP regulatory module, thereby influencing the infection and development of *M. incognita* in roots.

Apart from inhibiting miRNA expression, lncRNAs also impact the expression of related genes. For instance, lncRNA16397 promotes the expression of the glutaredoxin (GRX) gene, thereby modifying tomato resistance to late blight [25]. LncRNA33732 also enhances tomato resistance by inducing the expression of RBOH [10]. Additionally, rice lncRNA ALEX1 plays a regulatory role in plant defense pathways, and its overexpression plants can activate PR genes in the jasmonic acid pathway, thereby strengthening plant resistance to Xanthomonas oryzae [26]. Previous studies have indicated that TCP genes positively regulate jasmonic acid levels in plants and the jasmonic acid pathway plays a vital role in plant resistance to root-knot nematode infection [27]. In this study, lncRNA47258 induced the expression of the TCP gene. In tomato plants with silenced lncRNA47258, the accumulation of Solyc12g0141401.1, Solyc07g062681.1, and Solyc05g012844.1 decreased, and the level of jasmonic acid declined. The overexpression of lncRNA47258 and the target gene TCP verified that lncRNA47258 could regulate the jasmonic acid signaling pathway, increase the content of jasmonic acid in tomato roots, enhance the resistance of tomato to *M. incognita*, retard the development of *M. incognita*, and reduce the formation of root-knot number. The lncRNA47258/miR319b/TCP regulatory module participates in the process of tomato resistance to root-knot nematode induced by biocontrol bacterium Sneb821, and also discloses a novel regulatory mechanism of lncRNAs involved in root-knot nematode biological control.

## 5. Conclusions

The plant’s responses were previously considered as separated versus pathogens, but nematodes are often considered in between [28]. This study’s findings are summarized as follows: Sneb821 was demonstrated to interfere with the expression of non-coding RNA genes in tomato plants. It up-regulated the expression of lncRNA while inhibiting the expression of miRNA. Consequently, this regulation led to the up-regulation of the corresponding target genes and an increase in jasmonic acid content, enabling tomato plants to resist the infection of *M. incognita* and suppress the formation of root knots.

**Figure 7:**
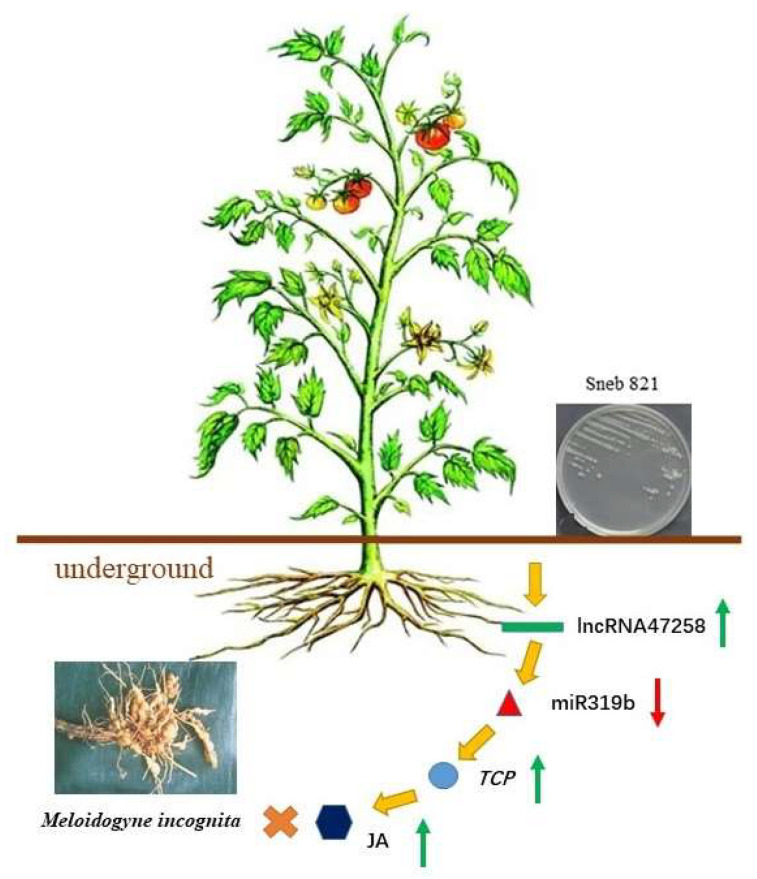
Model of resistance induced by Sneb821 to *M. incognita* in tomato. Note: Arrows indicate positive regulation, and blunt-ended bars indicate inhibition; the red arrows represent down-regulated expression and the green arrows represent up-regulated expression.

The differentially expressed miRNAs within the transcriptome were screened. In combination with previous reports indicating that miRNAs can respond to nematode infection, it was identified that multiple molecular regulatory modules, such as miR156/SPLs, miR319/TCP, miR390/ARFs, miR482/NBS-LRR, and miR396/GRFs, might be implicated in the induction and resistance processes. Thus, considering the in vivo ceRNA mechanism, lncRNAs capable of acting on miR156, miR482, and miR319 were selected for further in-depth investigation. LncRNA47258 was found to affect miR319b to modulate the content of tomato jasmonic acid, thereby inhibiting the impact of *M. incognita* (Figure 7). The results of this study hold significant importance for exploring the biological control mechanisms of plant defense, uncovering novel biomarkers for controlling plant nematode diseases, and devising new strategies for nematode disease control.

## Data Availability

The original contributions presented in the study are included in the article/Supplementary Material, further inquiries can be directed to the corresponding author.

## Supplementary Materials

The following supporting information can be downloaded at: https://www.mdpi.com/article/10.3390/pathogens14030256/s1, Figure S1: RT-PCR validation of tomato lncRNA. Figure S2: Construction of overexpressed tomato plants of pCAMBIA1302-lncRNA47258. Figure S3: Digestion verification of pMD20-T-lncRNA47258 and TRV2-lncRNA47258. Figure S4: Construction of overexpressed tomato plants of pCAMBIA1302-*Solyc07g062680.1*. Table S1: Primers used in this study.

## References

[1] Catani L., Manachini B., Grassi E., Guidi L., Semprucci F. (2023). Essential oils as nematicides in plant protection. Plants.

[2] Fujimoto T., Tomitaka Y., Abe H., Tsuda S., Futai K., Mizukubo T. (2011). Expression profile of jasmonic acid-induced genes and the induced resistance against the root-knot nematode in tomato plants after foliar treatment with methyl jasmonate. J. Plant Physiol..

[3] Wierzbicki A.T. (2012). The role of long non-coding RNA in transcriptional gene silencing. Curr. Opin. Plant Biol..

[4] Zhang H., Hu W., Hao J., Lv S., Wang C., Tong W., Wang Y., Wang Y., Liu X., Ji W. (2016). Genome-wide identification and functional prediction of novel and fungi-responsive lincRNAs in Triticum aestivum. BMC Genom..

[5] Zhang L., Wang M., Li N., Wang H., Qiu P., Pei L., Xu Z., Wang T., Gao E., Liu J. (2018). Long noncoding RNAs involve in resistance to *Verticillium dahliae*, a fungal disease in cotton. Plant Biotechnol. J..

[6] Qin T., Zhao H., Cui P. (2017). A nucleus-localized long non-coding RNA enhances drought and salt stress tolerance. Plant Physiol..

[7] Wu J.G., Yang R.X., Yang Z.R. (2017). ROS accumulation and antiviral defence control by microRNA528 in rice. Nat. Plants.

[8] Li W., Chen Y., Wang Y., Zhao J., Wang Y. (2022). Gypsy retrotransposon-derived maize 1 lncRNA GARR2 modulates gibberellin response. Plant J..

[9] Jiang C.H., Fan Z.H., Li Z.J., Niu D.D., Li Y., Zheng M.Z., Wang Q., Jin H.L., Guo J.H. (2020). Bacillus cereus AR156 triggers induced systemic resistance against *Pseudomonas syringae* pv. tomato DC3000 by suppressing miR472 and activating CNLs-mediated basal immunity in Arabidopsis. Mol. Plant Pathol..

[10] Cui J., Jiang N., Meng J., Yang G., Liu W., Zhou X., Ma N., Hou X., Luan Y. (2019). LncRNA33732-respiratory burst oxidase module associated with WRKY1 in tomato-Phytophthora infestans interactions. Plant J..

[11] Peng T., Liu X., Tian F., Xu H., Yang F., Chen X., Gao X., Lv Y., Li J., Pana Y. (2022). Functional investigation of lncRNAs and target cytochrome P450 genes related to spirotetramat resistance in *Aphis gossypii* Glover. Pest Manag. Sci..

[12] Yu J., Qiu K., Sun W., Yang T., Wu T., Song T., Zhang J., Yao Y., Tian J. (2022). A long non-coding RNA functions in high-light-induced anthocyanin accumulation in apple by activating ethylene synthesis. Plant Physiol..

[13] Zhang L., Liu J., Cheng J., Sun Q., Zhang Y., Liu J., Li H., Zhang Z., Wang P., Cai C. (2022). lncRNA7 and lncRNA2 modulate cell wall defense genes to regulate cotton resistance to Verticillium wilt. Plant Physiol..

[14] Zhang X., Shen J., Xu Q., Dong J., Song L., Wang W., Shen F. (2021). Long noncoding RNA lncRNA354 functions as a competing endogenous RNA of miR160b to regulate ARF genes in response to salt stress in upland cotton. Plant Cell Environ..

[15] Zheng Y., Wang Y., Ding B., Fei Z. (2017). Comprehensive transcriptome analyses reveal that potato spindle tuber viroid triggers genome-wide changes in alternative splicing, inducible trans-acting activity of phased secondary small interfering RNAs and immune responses. J. Virol..

[16] Wang J., Yang Y., Jin L., Ling X., Liu T., Chen T., Ji Y., Yu W., Zhang B. (2018). Re-analysis of long non-coding RNAs and prediction of circRNAs reveal their novel roles in susceptible tomato following TYLCV infection. BMC Plant Biol..

[17] Yang Z., Yang C., Wang Z., Yang Z., Chen D., Wu Y., Wu Y. (2019). LncRNA expression profile and ceRNA analysis in tomato during flowering. PLoS ONE.

[18] Yang F., Zhao D., Fan H.Y., Zhu X.F., Wang Y.Y., Liu X.Y., Duan Y.X., Xuan Y.H., Chen L.J. (2020). Functional analysis of long non-coding RNAs reveal their novel roles in biocontrol of bacteria-induced tomato resistance to *Meloidogyne incognita*. Int. J. Mol. Sci..

[19] Yang F., Ding L., Zhao D., Fan H.Y., Zhu X.F., Wang Y.Y., Liu X.Y., Duan Y.X., Xuan Y.H., Chen L.J. (2022). Identification and functional analysis of tomato microRNAs in the biocontrol bacterium Pseudomonas putida induced plant resistance to *Meloidogyne incognita*. Phytopathology.

[20] Zhao W.C., Li Z.L., Fan J.W., Hu C.L., Yang R., Qi X., Chen H., Zhao F.K., Wang S.H. (2015). Identification of jasmonic acid-associated microRNAs and characterization of the regulatory roles of the miR319/TCP4 module under root-knot nematode stress in tomato. J. Exp. Bot..

[21] Wang S.T., Sun X.L., Hoshino Y. (2014). MiR319 positively regulates cold tolerance by targeting OsPCF6 and OsTCP21 in rice. PLoS ONE.

[22] Wang J., Yu W., Yang Y. (2015). Genome-wide analysis of tomato long non-coding RNAs and identification as endogenous target mimic for microRNA in response to TYLCV infection. Sci. Rep..

[23] Jiang N., Meng J., Cui J., Sun G., Luan Y. (2018). Function identification of miR482b, a negative regulator during tomato resistance to Phytophthora infestans. Hortic. Res..

[24] Jiang N., Cui J., Shi Y.S., Yang G.L., Zhou X.X., Hou X.X., Meng J., Luan Y.S. (2019). Tomato lncRNA23468 functions as a competing endogenous RNA to modulate NBS-LRR genes by decoying miR482b in the tomato-Phytophthora infestans interaction. Hortic. Res..

[25] Cui J., Luan Y., Jiang N., Bao H., Meng J. (2017). Comparative transcriptome analysis between resistant and susceptible tomato allows the identification of lncRNA16397 conferring resistance to Phytophthora infestans by co-expressing glutaredoxin. Plant J..

[26] Yu Y., Zhou Y.F., Feng Y.Z., He H., Lian J.P., Yang Y.W., Lei M.Q., Zhang Y.C., Chen Y.Q. (2020). Transcriptional landscape of pathogen-responsive lncRNAs in rice unveils the role of ALEX1 in jasmonate pathway and disease resistance. Plant Biotechnol. J..

[27] Fan C.L., Hu L.N., Zhang Z. (2015). Jasmonic acid mediates tomato’s response to root knot nematodes. J. Plant Growth Regul..

[28] Gupta R., Mfarrej MF B., Xhemali B., Khan A., Nadeem H., Ahmad F. (2023). Metabolic responses of plants to *Meloidogyne* species parasitism: A review on molecular events and functions. J. King Saud Univ.-Sci..

