# Supplementary material for: The Tomato lncRNA47258-miR319b-TCP Module in Biocontrol Bacteria Sneb821 Induced Plants Resistance to Meloidogyne incognita
Source: Pathogens. 2025 Mar 5;14(3):256. doi: 10.3390/pathogens14030256 (PMC11945786; doi:10.3390/pathogens14030256)
Supplement: Supplementary file 1 [file pathogens-14-00256-s001.zip › Supplementary figure.pdf]

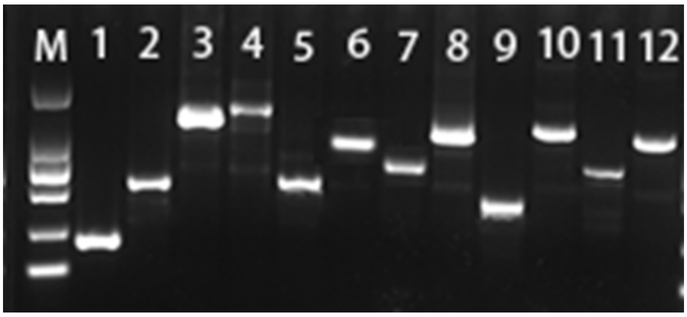

Figure S1. RT-PCR validation of tomato lncRNA. Note: Lane 1 is lncRNA18894, Lane 2 is lncRNA21563, Lane 3 is lncRNA24059, Lane 4 is lncRNA25797, Lane 5 is lncRNA35115, Lane 6 is lncRNA39939, Lane 7 is lncRNA8668, Lane 8 is lncRNA44664, Lane 9 is lncRNA45969, Lane 10 is lncRNA48734, Lane 11 is lncRNA51612 and Lane 12 is lncRNA7183.

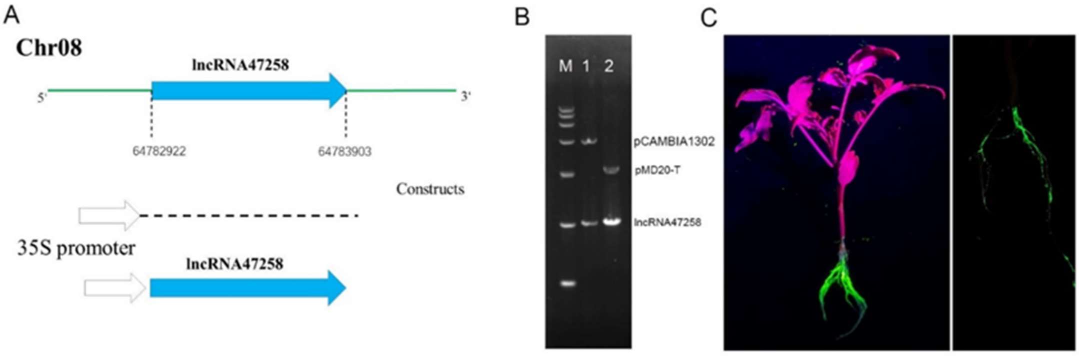

Figure S2. Construction of overexpressed tomato plants of pCAMBIA1302-lncRNA47258. Notes: (A) Schematic diagram of tomato genome lncRNA47258, (B) Electrophoretic diagram of pCAMBIA1302-lncRNA47258, (C) GFP fluorescence of tomato hairy roots.

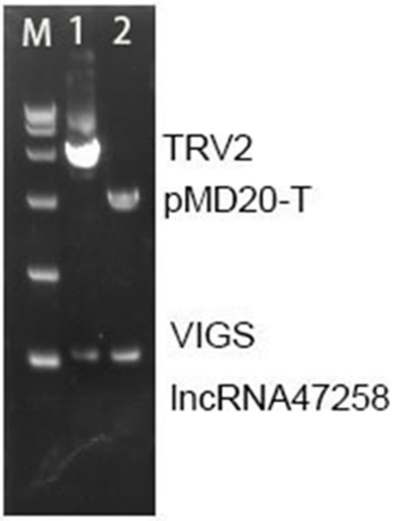

Figure S3. Digestion verification of pMD20-T-lncRNA47258 and TRV2-lncRNA47258.

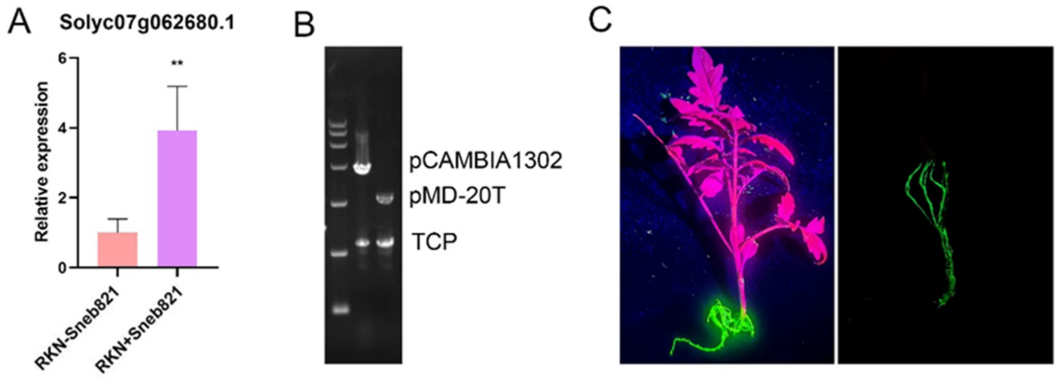

Figure S4. Construction of overexpressed tomato plants of pCAMBIA1302-*Solyc07g062680.1*. Note: (A) Relative expression of *Solyc07g062680.1* in RKN-Sneb821 and RKN+Sneb821, (B) Electrophoretic diagram of pCAMBIA1302-*Solyc07g062680.1*, (C) GFP fluorescence of tomato hairy roots.
